# Retraction: A Scoring Model Based on Neutrophil to Lymphocyte Ratio Predicts Recurrence of HBV-Associated Hepatocellular Carcinoma after Liver Transplantation

**DOI:** 10.1371/journal.pone.0220418

**Published:** 2019-07-23

**Authors:** The PLOS ONE Editors

Concerns have been raised that the transplants performed in the local context at the time of procedures reported in this article [1] may have involved organs/tissues procured from prisoners [2].

Details as to the donor sources and methods of obtaining informed consent from donors were not reported in [1], and when following up on these concerns the authors did not clarify these issues or the cause(s) of donor death in response to journal inquiries. International ethics standards call for transparency in organ donor and transplantation programs and clear informed consent procedures including considerations to ensure that donors are not subject to coercion.

The authors did not provide documentation when requested by the journal to confirm that the study had institutional ethics approval. They stated that all organs/tissues were obtained voluntarily for the transplant procedures, but did not provide documentation to support this claim or any further clarification regarding the informed consent procedure or causes of death for organ donors.

In addition, in response to journal requests about data availability, the authors did not provide underlying data supporting this study or comment on the availability of the data.

The authors noted that many of the original materials requested by the journal to clarify these issues are no longer available.

Owing to the lack of documentation to demonstrate this study had prospective ethical approval, insufficient reporting, unresolved concerns around the source of transplanted organs and whether they included organs from prisoners, and in compliance with international ethical standards for organ/tissue donation and transplantation, the *PLOS ONE* Editors retract this article.

The first author notified the journal that all authors agree with the retraction. The other authors either could not be reached or did not respond directly.
